# Fulminant Guillain-Barré syndrome showing severe pharyngeal-cervical-brachial weakness in the recovery phase: a case report

**DOI:** 10.1186/s12883-019-1376-5

**Published:** 2019-06-28

**Authors:** Yoshitsugu Nakamura, Mikiko Motoki, Takahiko Hirose, Takafumi Hosokawa, Shimon Ishida, Shigeki Arawaka

**Affiliations:** Department of Internal Medicine IV, Division of Neurology, Osaka Medical College, 2-7 Daigakumachi, Takatsukishi, Osaka, 569-8686 Japan

**Keywords:** Guillain-Barré syndrome, Pharyngeal-cervical-brachial weakness, Fulminant, Anti-GT1a antibody, Case report

## Abstract

**Background:**

Fulminant Guillain-Barré syndrome (GBS) is characterized clinically by rapid progression of severe symptoms, such as the absence of brainstem reflexes, complete tetraplegia and respiratory arrest. The clinical course of fulminant GBS remains unclear. Here, we report a patient with fulminant GBS, who showed severe weakness of the pharyngeal-cervical-branchial (PCB) area in the recovery phase.

**Case presentation:**

A 38-year-old man rapidly developed fulminant GBS. In blood examination, he was positive for a broad range of anti-ganglioside antibodies, including anti-GQ1b, GT1a, GT1b, GD1a, GD1b and GD3 IgG antibodies. We performed immunosuppressive therapies using intravenous immunoglobulin and intravenous methylprednisolone. Although disturbance of consciousness and weakness of the distal upper and lower limbs improved gradually, weakness of the oropharynx, neck, and proximal upper limbs were resistant to these therapies. Anti-GT1a IgG antibodies remained persistently positive. Consequently, mechanical ventilation and tube feeding were required for 7 and 10 months, respectively. Two years later, weakness of the proximal upper limbs and mild respiratory dysfunction remained as sequelae.

**Conclusion:**

Anti-GT1a IgG antibodies are known to be detected in patients with the PCB variant of GBS. In fulminant GBS, the persistent presence of anti-GT1a IgG antibodies may be associated with occurrence of severe PCB-like weakness in the recovery phase.

## Background

The pharyngeal-cervical-brachial (PCB) variant of Guillain-Barré syndrome (GBS) is characterized by rapidly progressive weakness of oropharyngeal and cervicobrachial areas with areflexia in the upper limbs [1, 2]. The PCB variant is part of the clinical spectrum containing GBS, Fisher syndrome and Bickerstaff brainstem encephalitis [1]. Additionally, a patient with GBS who initially undergoes typical clinical course occasionally shows PCB-like weakness in the recovery phase [3]. It is unclear as to the relationship between the PCB variant and fulminant GBS [4, 5]. Here, we report a patient with fulminant GBS showing severe PCB-like weakness and the persistent presence of anti-GT1a IgG antibodies in the recovery phase.

## Case presentation

**Fig. 1:**
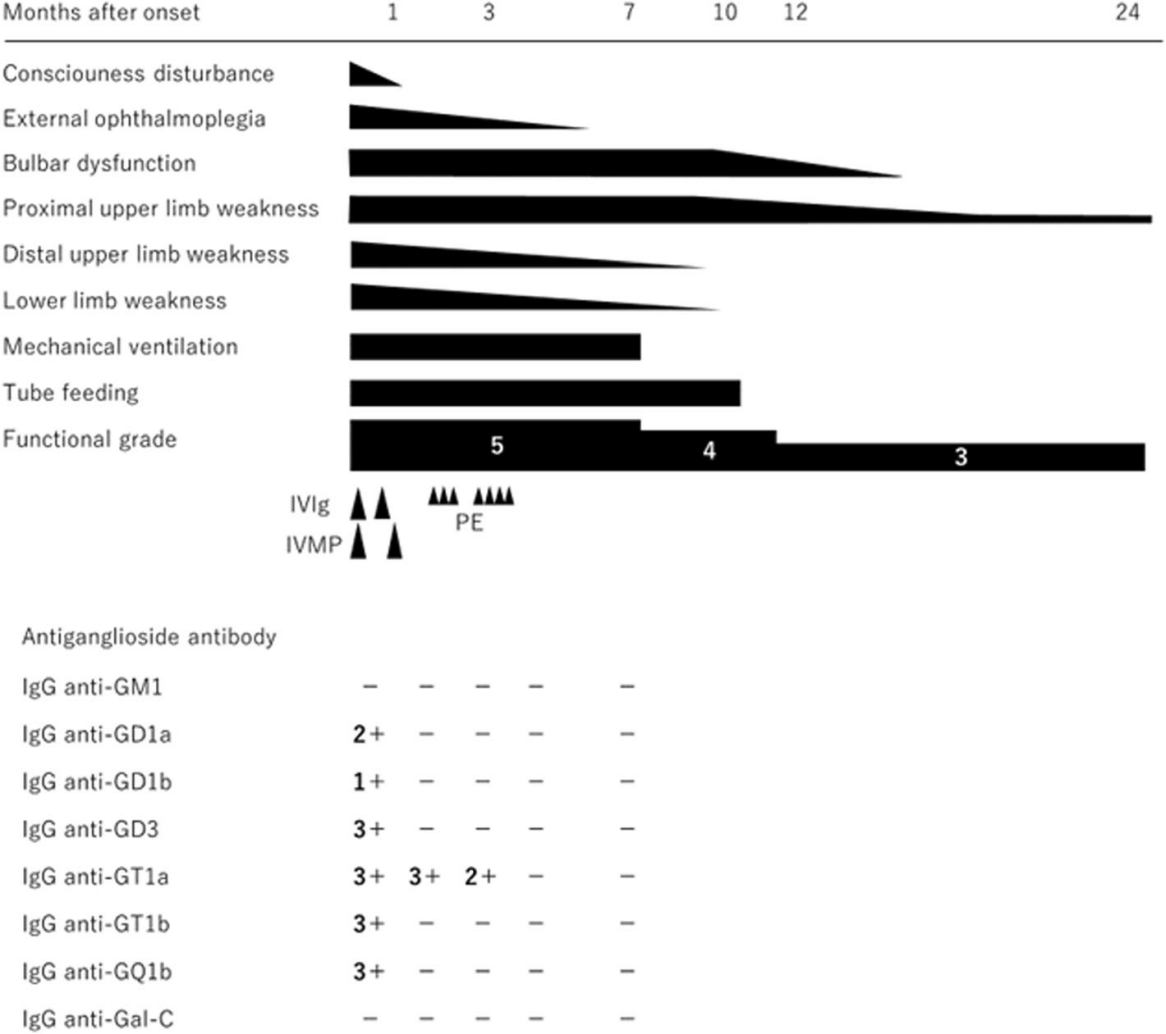
Clinical course and change in the titers of anti-ganglioside IgG antibodies. Anti-ganglioside IgG antibodies of the present patient were measured by enzyme-linked immunosorbent assay. According to optical density, the titers were determined semi-quantitively as - to 4+. Patient disability was evaluated on the Functional grade: grade 6, dead; grade 5, requires assisted ventilation; grade 4, bed bound; grade 3, able to walk 5 m with aid; grade 2, able to walk 5 m independently; grade 1, minimal signs and symptoms, able to run; grade 0, normal. Abbreviations: IVIg = intravenous immunoglobulin; IVMP = intravenous methylprednisolone; PE = plasma exchange

A 38-year-old man was aware of bilateral lower limb weakness 3 days after upper respiratory infection. The next day, he showed disturbance of consciousness and bilateral upper limb weakness. Two days after onset, he showed respiratory failure and needed support by mechanical ventilation. The patient was admitted to our hospital. At admission, he showed bradycardia (heart rate was 40 beats per minute). In neurological examination under no sedation, he showed no response to painful and visual stimuli. Light reflex was bilaterally dull though pupil diameter was 5 mm. No voluntary ocular and facial movements were observed. Oculocephalic, corneal, gag and cough reflexes were absent. He showed complete flaccid tetraplegia with areflexia in all limbs. Babinski reflex was negative. At 13 days after onset, cerebrospinal fluid examination revealed a normal cell count at 4 /μL, but protein levels increased to 98.5 mg/dL. At 21 days after onset, the nerve conduction study showed that compound muscle and sensory nerve action potentials decreased from the distal portion in upper limb nerves, and distal latencies and nerve conduction velocities were normal in all nerves tested. These findings were electrophysiologically consistent with the pattern of axonal damage in peripheral nerves (Table 1). Brain MRI showed no intracranial abnormal signals on diffusion-, T1-, T2- and fluid-attenuated inversion recovery-weighted images. Spinal MRI also showed no intramedullary abnormal signals. Auditory brain stem response was normal. Various anti-ganglioside antibodies were detected in laboratory examinations. Anti-GQ1b, GT1a, GT1b, GD1a, GD1b and GD3 IgG antibodies were positive (Fig. 1). These data confirmed the diagnosis of GBS. At 4 days after onset, we started to administrate intravenous immunoglobulin (IVIg) at a daily dose of 0.4 g/kg for five days and intravenous methylprednisolone (IVMP) at a daily dose of 1000 mg for three days. At 22 days after onset, we repeated IVIg, followed by IVMP (Fig. 1). Disturbance of consciousness, eye symptoms and weakness of the distal upper and lower limbs improved gradually, whereas severe PCB-like weakness of the oropharynx, neck, and proximal upper limbs remained. Anti-GQ1b, GT1b, GD1a, GD1b and GD3 IgG antibodies were turned to be negative, but anti-GT1a IgG antibodies remained positive. We added plasma exchange (PE) three times from 39 days after onset. However, the PCB-like weakness did not improve, muscle atrophy of limbs became apparent, and anti-GT1a IgG antibodies persistently positive. We further performed PE four times from 68 days after onset. Although anti-GT1a IgG antibodies decreased, severe PCB-like weakness did not ameliorate. Consequently, mechanical ventilation and tube feeding was required for 7 and 10 months, respectively. At 18 months after onset, in the nerve conduction study, the decrease in compound muscle action potentials in upper limb nerves was persistently observed (Table 1). Two years later, he could walk using assistance, but weakness of the proximal upper limbs remained as sequelae (Fig. 1). Additionally, his pulmonary dysfunction failed to improve fully as vital capacity decreased to 71.0%.

**Table 1:** Summary of the nerve conduction study in the acute and recovery phases

Acute phase	Motor nerves				
	Nerve	Side	Distal latency (ms)	Distal amplitude (mV)	Conduction velocity (m/s)
	Median	Right	4.1	0.36	58.5
	Ulnar	Right	3	0.19	53.5
	Tibial	Right	3.3	4.5	46.7
	Sensory nerves				
	Nerve	Side	Latency (ms)	Amplitude (μV)	Conduction velocity (m/s)
	Median	Right	2.5	3.4	55.6
	Ulnar	Right	2.8	2	49.3
	Sural	Right	2.8	13.9	50.7
Recovery phase	Motor nerves				
	Nerve	Side	Distal latency (ms)	Distal amplitude (mV)	Conduction velocity (m/s)
	Median	Right	3.4	5.2	62.2
	Ulnar	Right	2.8	5.1	63.8
	Tibial	Right	4	22	57.5
	Sensory nerves				
	Nerve	Side	Latency (ms)	Amplitude (μV)	Conduction velocity (m/s)
	Median	Right	2	22.1	68.6
	Ulnar	Right	2.5	9.5	56.9
	Sural	Right	2.9	33.9	54.7

In the acute phase, compound muscle and sensory nerve action potentials decreased from the distal portion in upper limb nerves as compared with those in lower limb nerves. In all nerves tested, distal latencies and nerve conduction velocities were normal. These findings suggested axonal damage in peripheral nerves. In the recovery phase, compound muscle action potentials in upper limb nerves persistently decreased

## Discussion and conclusions

Fulminant GBS is characterized by rapidly showing severe symptoms, such as the absence of brainstem reflexes, complete tetraplegia and respiratory arrest [5]. Fulminant GBS is expressed as a brain death-like case. The present patient showed disturbance of consciousness, respiratory failure, and absent multiple brainstem reflexes and tetraplegia with areflexia of limbs within three days after onset of the first symptoms. This severity seemed to be consistent with fulminant GBS. In the recovery phase, although disturbance of consciousness and weakness of distal upper and lower limbs improved, severe PCB-like weakness of the oropharynx, neck, and proximal upper limbs were persistently observed. Additionally, after IVIg and IVMP treatments, a majority of anti-ganglioside antibodies were disappeared, but anti-GT1a IgG antibodies remained persistently positive. Anti-GT1a IgG antibodies are reported to recognize the glycolipid fraction of glossopharyngeal and vagal nerves [2]. Anti-GT1a IgG antibodies are detected in patients with PCB [1–3, 6, 7]. Anti-GT1a IgG antibodies may be associated with bulbar dysfunction [2, 6, 7]. Additionally, anti-GT1a IgG antibodies are proposed to be associated with multiple cranial nerve palsy [8] and severe generalized muscle weakness and atrophy [6]. In the present patient, it was unclear whether the PCB variant of GBS rapidly transferred to fulminant one. However, severe PCB-like weakness in the recovery phase was concomitant with the persistent presence of anti-GT1a IgG antibodies. This finding suggests that the persistent presence of anti-GT1a IgG antibodies is associated with the occurrence of severe PCB-like weakness in the recovery phase of fulminant GBS. To validate this idea, it is necessary to further accumulate patients with fulminant GBS positive for anti-GT1a IgG antibodies and analyze the relation between the presence of anti-GT1a IgG antibodies and the PCB-like symptom.

## Data Availability

All primary data supporting the findings of this study are available within this article.

## Abbreviations

GBS: Guillain-Barré syndrome
IVIg: Intravenous immunoglobulin
IVMP: Intravenous methylprednisolone
PCB: Pharyngeal-cervical-brachial
PE: Plasma exchange
